# Template-Free Synthesis of Sb_2_S_3_ Hollow Microspheres as Anode Materials for Lithium-Ion and Sodium-Ion Batteries

**DOI:** 10.1007/s40820-017-0165-1

**Published:** 2017-10-31

**Authors:** Jianjun Xie, Li Liu, Jing Xia, Yue Zhang, Min Li, Yan Ouyang, Su Nie, Xianyou Wang

**Affiliations:** 1 National Base for International Science and Technology Cooperation, National Local Joint Engineering Laboratory for Key Materials of New Energy Storage Battery, Hunan Province Key Laboratory of Electrochemical Energy Storage and Conversion, School of Chemistry, Xiangtan University, Xiangtan, 411105 People’s Republic of China; 2 Key Laboratory of Advanced Energy Materials Chemistry (Ministry of Education), Nankai University, Tianjin, 300071 People’s Republic of China

**Keywords:** Sb_2_S_3_, Hollow microspheres, Anode material, Lithium-ion batteries, Sodium-storage property

## Abstract

Hierarchical Sb_2_S_3_ hollow microspheres assembled by nanowires have been successfully synthesized by a simple and practical hydrothermal reaction. The possible formation process of this architecture was investigated by X-ray diffraction, focused-ion beam-scanning electron microscopy dual-beam system, and transmission electron microscopy. When used as the anode material for lithium-ion batteries, Sb_2_S_3_ hollow microspheres manifest excellent rate property and enhanced lithium-storage capability and can deliver a discharge capacity of 674 mAh g^−1^ at a current density of 200 mA g^−1^ after 50 cycles. Even at a high current density of 5000 mA g^−1^, a discharge capacity of 541 mAh g^−1^ is achieved. Sb_2_S_3_ hollow microspheres also display a prominent sodium-storage capacity and maintain a reversible discharge capacity of 384 mAh g^−1^ at a current density of 200 mA g^−1^ after 50 cycles. The remarkable lithium/sodium-storage property may be attributed to the synergetic effect of its nanometer size and three-dimensional hierarchical architecture, and the outstanding stability property is attributed to the sufficient interior void space, which can buffer the volume expansion.

**Electronic supplementary material:**

The online version of this article (doi:10.1007/s40820-017-0165-1) contains supplementary material, which is available to authorized users.

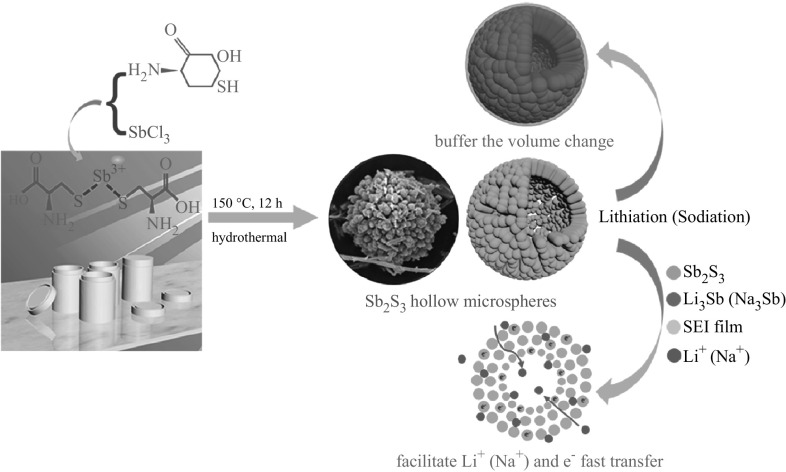

## Highlights

- Sb_2_S_3_ hollow microspheres have been successfully synthesized by a simple hydrothermal reaction using SbCl_3_ and l-cysteine as raw materials without adding any surfactants.
- The novel architecture combines the merits of nanometer size, hollow interior, and 3D hierarchical structure.
- The material presents remarkable cycling performance and outstanding rate capability in lithium-ion batteries and also exhibits superior sodium-storage capabilities in sodium-ion batteries.

## Introduction

Owing to the numerous inherent advantages of lithium-ion batteries (LIBs), they have been generally applied in many fields and display good prospect in large-scale energy-storage systems [1–5]. However, sodium-ion batteries (NIBs) have attracted much attention as a potential substitute to LIBs [6–8]. In recent years, there have been successful studies into the development of cathode materials for LIBs and NIBs [9, 10]. Therefore, many researchers have focused on exploring potential suitable anode materials for future LIBs and NIBs [11–15]. Among the many suitable anode materials for LIBs and NIBs, antimony sulfide (Sb_2_S_3_) is a highly anisotropic semiconductor that crystallizes with a layered structure, and it has received significant attention owing to its high theoretical specific capacity (947 mAh g^−1^) and superior lithium/sodium-storage performance [14–16]. For instance, Zhou et al. [17] demonstrated Sb_2_S_3_ nanorod bundles with a fine cyclability of 614 mAh g^−1^ at a current density of 100 mA g^−1^ after 30 cycles, as well as an excellent rate capability of 400 mAh g^−1^ at a current density of 500 mA g^−1^ in LIBs. Yi et al. [18] reported Sb_2_S_3_ with a discharge capacity of 548 mAh g^−1^ at a current density of 100 mA g^−1^ after 100 cycles in LIBs. Discharge capacities of 659.7, 564.9, 434.2, and 300.5 mAh g^−1^ are achieved at current densities of 100, 200, 500, and 1000 mA g^−1^, respectively. Denis et al. [19] exhibited the cyclic property of commercial Sb_2_S_3_ at a current density of 50 mA g^−1^ in NIBs. During the initial 10 cycles, the discharge capacity of commercial Sb_2_S_3_ increases from 337 to 419 mAh g^−1^. However, after 50 cycles, it decreases to less than 200 mAh g^−1^, which is under 50% of the maximum capacity. Pan et al. [20] reported the template-free synthesis of Sb_2_S_3_ microtubes, whose respective initial discharge and charge capacities are 910 and 400 mAh g^−1^ at a current density of 100 mA g^−1^ in NIBs, respectively. After 20 cycles, the capacity remains at 201 mAh g^−1^.

Over the past few decades, many studies have been devoted to the exploration of Sb_2_S_3_ with various particle sizes, morphologies, coating, as well as composites for enhancing rate capability and cycling stability. Recently, the design and synthesis of three-dimensional (3D) hierarchical architecture Sb_2_S_3_ materials have attracted considerable attention, owing to their capability to effectively improve electrochemical performance by enlarging the contact surface area between the electrolyte and electrode, reducing the transport pathway of electrons and Li^+^/Na^+^, as well as accommodating the volume expansion [21–25].

Here, Sb_2_S_3_ hollow microspheres have been effectively synthesized by a straightforward hydrothermal reaction employing l-cysteine and SbCl_3_ as raw materials without adding any surfactants. This novel architecture combines the merits of hollow and 3D hierarchical structures. As expected, the Sb_2_S_3_ hollow microspheres exhibit superior lithium/sodium-storage capacity and outstanding rate property.

## Experimental Section

### Material Synthesis

The Sb_2_S_3_ samples were prepared using a hydrothermal reaction. To obtain Sb_2_S_3_ hollow microspheres, 3 mmol l-cysteine was dissolved in 30 mL distilled water under magnetic stirring for 15 min to obtain a colorless solution. Then, 30 mL distilled water and 2 mmol SbCl_3_ were systematically poured into the beaker under stirring for 35 min to produce a milky suspension liquid. Afterward, the above solution was transferred into an 80-mL Teflon-lined stainless-steel autoclave and then maintained at 150 °C for 12 h. The Sb_2_S_3_ was gathered and washed several times with absolute ethyl alcohol and deionized water, and dried at 80 °C for 5 h in vacuum. Comparable samples were prepared using a similar procedure at alter hydrothermal reaction temperatures of 120 °C and 180 °C, respectively. These three samples are denoted as Sb_2_S_3_-120, Sb_2_S_3_-150, and Sb_2_S_3_-180 according to the variation of reaction temperature.

### Electrochemical Measurements

The work electrode was prepared by mixing the carboxymethyl cellulose Na salt (CMC), acetylene black, and active material in the mass ratio of 1:1:3, after which the viscosity was varied with distilled water and magnetic stirring overnight. The loading mass of the active material is around 1 mg cm^−2^. For LIBs, the testing batteries were assembled with the working electrode already prepared, and with Celgard 2300 film as the separator, lithium metal as the reference/counter electrode, and 1 M LiPF_6_ in dimethyl carbonate (DMC)/ethylene carbonate (EC) (1:1 v/v) as the electrolyte. However, NIBs were assembled with glass fiber film (Whatman GF/D) as the separator, metal sodium as the reference/counter electrode, and 1 M NaClO_4_ in ethylene carbonate (EC)/propylene carbonate (PC) (1:1 v/v) as the electrolyte. All batteries were fabricated in an argon-filled glove box with H_2_O and O_2_ values below 1 ppm. The charge/discharge measurements of the Sb_2_S_3_ electrodes were taken on a Neware battery test system at room temperature.

### Material Characterization

The morphology, structure, and interplanar distance of the samples were verified using a focused-ion beam-scanning electron microscope dual-beam system (FIB-SEM, FEI Helios Nanolab 600i), transmission electron microscopy (TEM), and high-resolution transmission electron microscopy (HRTEM JEOL JEM-2100F). X-ray diffraction (XRD) data were obtained using a Rigaku D/MAX-2500 power diffractometer for 2*θ* = 10°–70°. Cyclic voltammograms (CV) and electrochemical impedance spectroscopy (EIS) tests were executed on a Zahner Zennium electrochemical workstation.

## Results and Discussion

### Characterization of the As-Prepared Sb_2_S_3_

**Fig. 1:**
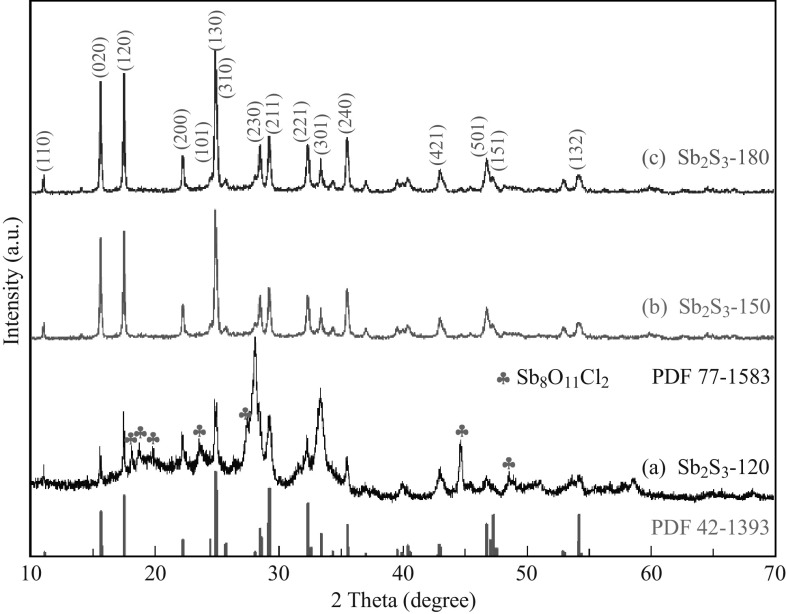
XRD patterns of as-prepared **a** Sb_2_S_3_-120, **b** Sb_2_S_3_-150, and **c** Sb_2_S_3_-180 samples

The phase composition and crystal structure of the as-prepared Sb_2_S_3_ samples were identified by XRD, as illustrated in Fig. 1. The diffraction peaks of Sb_2_S_3_-120 can be indexed as orthorhombic Sb_2_S_3_ phase (space group: Pbnm) and monoclinic Sb_8_O_11_Cl_2_ phase (space group: C2m), which are in accordance with the standard date files PDF 42-1393 and PDF 77-1583, respectively. However, all diffraction peaks of Sb_2_S_3_-150 and Sb_2_S_3_-180 agree well with the standard diffraction patterns of orthorhombic Sb_2_S_3_ (PDF 42-1393, space group: Pbnm), and no other impure phases were detected. Furthermore, the sharp and narrow peaks indicate that these two samples are highly crystalline.

**Fig. 2:**
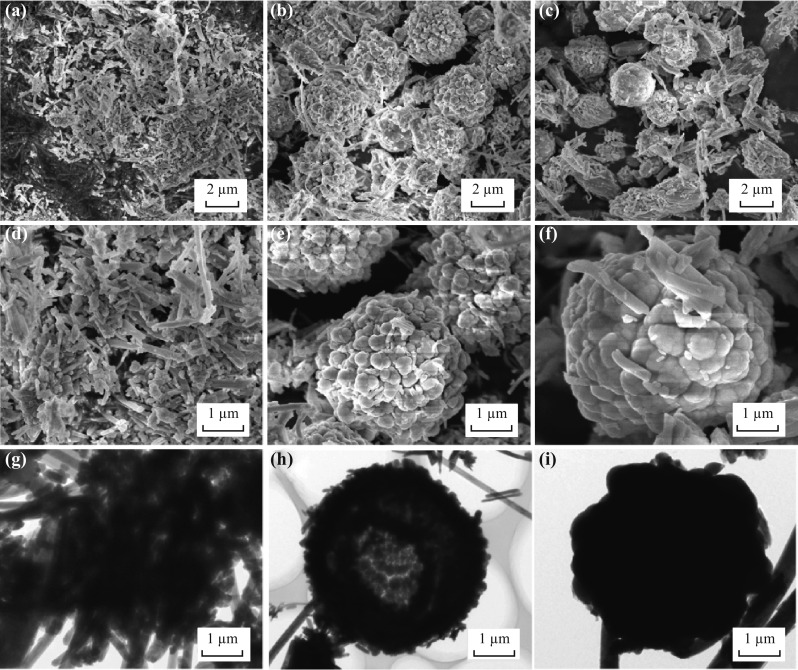
**a**-**f** FIB-SEM and **g**-**i** TEM images of **a**, **d**, **g** Sb_2_S_3_-120, **b**, **e**, **h** Sb_2_S_3_-150, and **c**, **f**, **i** Sb_2_S_3_-180 samples

The morphologies and sizes of these three samples were characterized by FIB-SEM and TEM, as shown in Fig. 2. The feature images of the prepared Sb_2_S_3_-120 sample are exhibited in Fig. 2a, d, g, and have numerous nanowires with diameters ranging from 80 to 100 nm. Moreover, densely aggregated primary nanowires can be clearly observed. When the reaction temperature was increased to 150 °C (Sb_2_S_3_-150), multiple 2–3 μm microsphere structures were obtained (Fig. 2b, e, h). More interestingly, by dissecting a randomly selected particle of the Sb_2_S_3_-150 sample, the FIB-SEM images reveal their hollow interior, and the 3D hollow microsphere structures are in fact built from irregularly shaped nanowires (Fig. S1). It has been reported that this 3D hierarchical hollow architecture will facilitate electrolyte percolation and increase the contact surface area between the electrolyte and electrode [26–30]. Besides, owing to the multiple levels of the structure, this structure can provide both large surface and firm stability [22, 30]. With a further increase in the reaction temperature to 180 °C, Sb_2_S_3_-180 shows rough solid spheres (Fig. 2c, f, i). It is evident that the reaction temperature has a significant effect on the morphology of the materials.

**Fig. 3:**
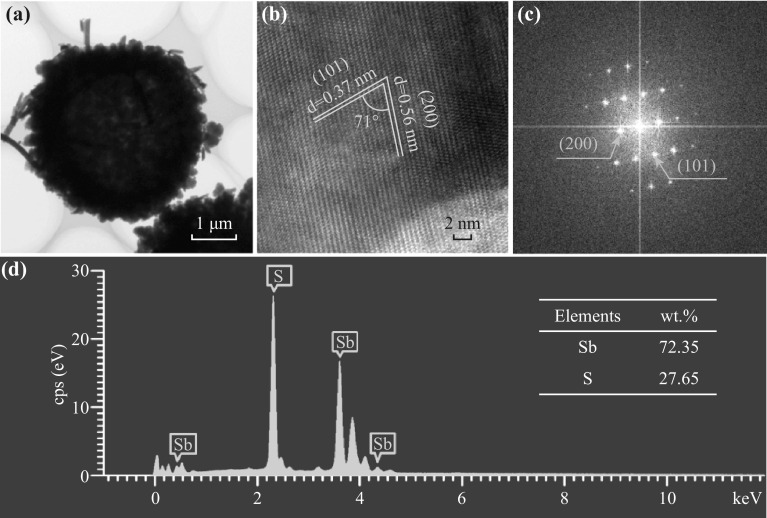
**a** TEM image of random selected a particle of Sb_2_S_3_-150 sample. **b** HRTEM image of Sb_2_S_3_-150 sample taken from **a**. **c** FFT pattern of Sb_2_S_3_-150 sample. **d** EDS spectrum of Sb_2_S_3_-150 sample

To evaluate the structure and element distribution of the Sb_2_S_3_-150 sample, TEM image of a randomly selected particle of Sb_2_S_3_-150 was acquired and is shown in Fig. 3a. It is clearly seen that the Sb_2_S_3_-150 microsphere has a hollow interior structure. The HRTEM image of the Sb_2_S_3_-150 sample shown in Fig. 3b. Figure 3b exhibits clear lattice fringes and showed that the interplanar distance between the adjacent lattices is 0.37 and 0.56 nm, corresponding to the (101) and the (200) planes of orthorhombic Sb_2_S_3_ (space group: Pbnm), respectively. The fast Fourier transform (FFT) pattern (Fig. 3c) reveals that the observed reflections of the Sb_2_S_3_-150 sample are uniquely indexed in the orthorhombic Sb_2_S_3_ (space group: Pbnm). All of these are in accordance with the XRD result. The element distribution of the Sb_2_S_3_-150 sample was studied using the EDS spectrum in Fig. 3d and demonstrated that the sample is composed of Sb and S elements, while the weight ratio of Sb to S is estimated to be 72.35:27.65. According to the calculation result, the atomic ratio of Sb to S is very close to 2:3, which further confirms that the product is orthorhombic Sb_2_S_3_ (space group: Pbnm).

Based on the above experimental results, the generation of hollow microsphere structures may be ascribed to the Ostwald ripening process, which has been extensively studied by many researchers in recent years [31, 32]. The possible mechanism involved in the formation of hollow microsphere structures is as follows: Sb_2_S_3_ nanoparticles are produced at the preliminary stages and interconnected to form nanowires. Then, a mass of nanowires is densely aggregated and coarsen. With increasing reaction temperature, these nanowires are stacked to generate hollow microspheres.

### Electrochemical Studies in Lithium-Ion Batteries

**Fig. 4:**
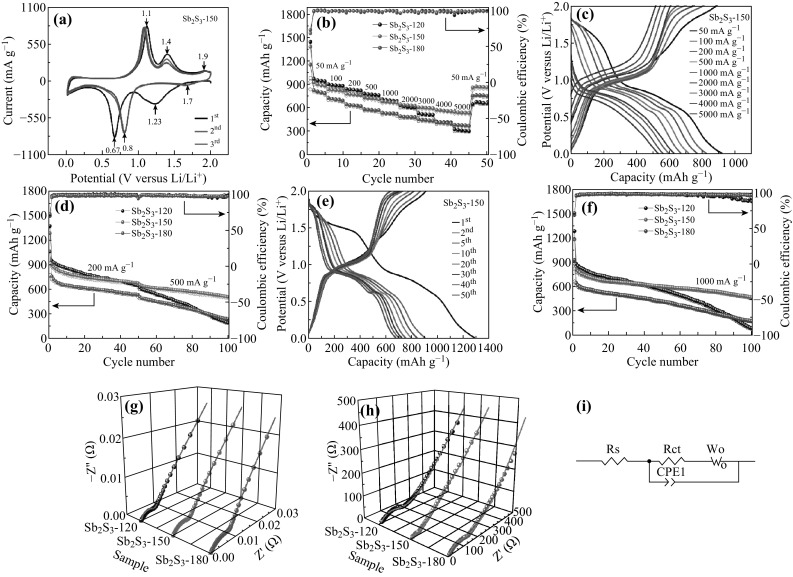
Excellent lithium-storage performance of as-prepared Sb_2_S_3_ electrodes. **a** CV curves of Sb_2_S_3_-150 electrode for the first three cycles at a scan rate of 0.1 mV s^−1^. **b** Rate performance of Sb_2_S_3_-120, Sb_2_S_3_-150, and Sb_2_S_3_-180 electrodes at various current densities from 50 to 5000 mA g^−1^. **c** Selected charge/discharge voltage profiles of Sb_2_S_3_-150 electrode at different current densities (increased from 50 to 5000 mA g^−1^). **d** Cycling performance of Sb_2_S_3_-120, Sb_2_S_3_-150, and Sb_2_S_3_-180 electrodes at different current densities. **e** Selected charge/discharge voltage profiles of Sb_2_S_3_-150 electrode at a current density of 200 mA g^−1^. **f** Cycling performance of Sb_2_S_3_-120, Sb_2_S_3_-150, and Sb_2_S_3_-180 electrodes at a current density of 1000 mA g^−1^. **g** Three-dimensional Nyquist plots of Sb_2_S_3_-120, Sb_2_S_3_-150, and Sb_2_S_3_-180 electrodes at the open-circuit voltage. **h** Three-dimensional Nyquist plots of Sb_2_S_3_-120, Sb_2_S_3_-150 and Sb_2_S_3_-180 electrodes at a current density of 500 mA g^−1^ after 50 cycles. **i** The equivalent circuit model

The cyclic voltammograms (CV) and galvanostatic charge/discharge measurements were taken to assess the electrochemical capability of Sb_2_S_3_-120, Sb_2_S_3_-150, and Sb_2_S_3_-180 as anode materials for LIBs. Figure 4a displays CV curves of the Sb_2_S_3_-150 electrode for the first three cycles at a scan rate of 0.1 mV s^−1^ in the voltage range of 0.01–2.0 V (vs. Li/Li^+^). During the first scan, the cathodic peaks are located at 1.23 and 0.67 V corresponding to the conversion reaction of Li with Sb_2_S_3_ (Eq. 1) and alloying reaction of Li with Sb (Eq. 2), respectively. The anodic peaks that are centered at 1.1, 1.4, and 1.9 V are related to the de-alloying reaction (reverse reaction of Eq. 2) and the formation of Sb_2_S_3_ (reverse reaction of Eq. 1) [14, 15]. As with the Sb_2_S_3_-120 electrode (Fig. S2a) and Sb_2_S_3_-180 electrode (Fig. S2b), the CV curve of Sb_2_S_3_-150 for the first cathodic scan is different from those of subsequent scans, indicating the presence of an activation process in the initial discharge process [19, 33–35]. From the second scanning, the stable reduction peak of the conversion reaction is located at 1.7 V and the alloying reaction is located at 0.8 V. The oxidation peaks are stably located, and all the peaks are overlapped, indicating the good electrochemical stability of Sb_2_S_3_-150. Figure S2a shows the CV scan curves of the Sb_2_S_3_-120 electrode. During the first scan, the cathodic peaks are located at 1.1 and 1.4 V, and the anodic peaks are located at 0.67 and 1.2 V. The CV scan curves of the Sb_2_S_3_-180 electrode are exhibited in Fig. S2b. In the first scan, the conversion reaction has anodic and cathodic peaks that are located at 1.85 and 1.2 V, respectively. The anodic peaks of alloying reaction are located at 1.1 and 1.4 V; the cathodic peak is 0.68 V. Remarkably, a polarization overpotential of 0.2 V for the conversion reaction is observed in Sb_2_S_3_-150 (0.35 V for Sb_2_S_3_-180), indicating that the voids inside the hollow structure can lead to smaller overpotential and faster reaction kinetics at the electrode surface [10].

1 $${\text{Conversion\,reaction:}}{\text{ Sb}}_{2} {\text{S}}_{3} + \, 6{\text{Li}}^{ + } + \, 6{\text{e}}^{ - } { \leftrightharpoons }3{\text{Li}}_{2} {\text{S }} + \, 2{\text{Sb}}$$

2 $${\text{Alloying\,reaction:}} \, 2{\text{Sb }} + \, 6{\text{Li}}^{ + } + \, 6{\text{e}}^{ - } { \leftrightharpoons }2{\text{Li}}_{3} {\text{Sb}}$$

The rate capability of Sb_2_S_3_-120, Sb_2_S_3_-150, and Sb_2_S_3_-180 electrodes was evaluated at various current densities from 50 to 5000 mA g^−1^ in the voltage range of 0.01–2.0 V (vs. Li/Li^+^), and the samples were tested for five cycles at each current density. From Fig. 4b, it is observed that Sb_2_S_3_-150 shows the best rate capability. When cycled at different current densities of 50, 100, 200, 500, 1000, 2000, 3000, 4000, and 5000 mA g^−1^, the Sb_2_S_3_-150 electrode presents discharge capacities of 1379, 831, 774, 727, 677, 639, 601, 568, and 541 mAh g^−1^, respectively. More importantly, the capacity could be quickly recovered after the current density was reduced to 50 mA g^−1^, indicating excellent rate performance. The Sb_2_S_3_-120 electrode exhibits a higher discharge capacity than Sb_2_S_3_-150 at low current densities in the first 20 cycles. Then, it shows a similar specific capacity as Sb_2_S_3_-150 at current densities of 1000 and 2000 mA g^−1^. However, as the current densities increase to 3000, 4000, and 5000 mA g^−1^, Sb_2_S_3_-150 exhibits a higher specific capacity than Sb_2_S_3_-120. Sb_2_S_3_-180 shows a much lower specific capacity than Sb_2_S_3_-150 at various current densities. The superior rate capability of Sb_2_S_3_-150 is attributed to its unique hollow microsphere architecture, where the nanosize properties combine with microsize properties to facilitate the rapid transfer of Li^+^ and electrons [36].

Figure 4c presents the selected charge/discharge voltage profiles of the Sb_2_S_3_-150 electrode at different current densities in the voltage range of 0.01–2.0 V (vs. Li/Li^+^). The corresponding profiles of Sb_2_S_3_-120 and Sb_2_S_3_-180 are shown in Fig. S3. Compared with Sb_2_S_3_-120 and Sb_2_S_3_-180, Sb_2_S_3_-150 shows a minimal increase in polarization with an increase in current density.

Figure 4d reveals the cycling performance of Sb_2_S_3_-120, Sb_2_S_3_-150, and Sb_2_S_3_-180 at different current densities in the voltage range of 0.01–2.0 V (vs. Li/Li^+^). It can be seen that their initial discharge/charge capacities of 1369/958, 1281/911, and 1058/767 mAh g^−1^ correspond to initial coulombic efficiencies (CE) of 69%, 71%, and 72.5%, respectively. The major contribution to the additional capacity is attributed to the inevitable formation of solid electrolyte interphase (SEI) film [37, 38]. This phenomenon is normally observed in various metal oxides or sulfide-based anode materials [39–41]. After the first few charge/discharge cycles, the CE rapidly increases to about 99% for all three electrodes. It is determined that the discharge capacity of the Sb_2_S_3_-150 sample can be kept at about 674 mAh g^−1^ (643 mAh g^−1^ for Sb_2_S_3_-120 and 532 mAh g^−1^ for Sb_2_S_3_-180) at a current density of 200 mA g^−1^ after 50 cycles. When the current density increased to 500 mA g^−1^, there was a slight decrease in the capacity, and it was maintained at about 506 mAh g^−1^ (189 mAh g^−1^ for Sb_2_S_3_-120 and 234 mAh g^−1^ for Sb_2_S_3_-180) after 100 cycles. The Sb_2_S_3_-120 electrode has a higher discharge capacity, but the cycling stability is inferior. The Sb_2_S_3_-150 with a hollow microsphere structure that is used as anode materials for LIBs has a remarkable cycling stability, even though it does not possess any conducting polymer or carbon.

Figure 4e shows the charge/discharge voltage profiles of the Sb_2_S_3_-150 electrode at a current density of 200 mA g^−1^ in the voltage range of 0.01–2.0 V (vs. Li/Li^+^). During the initial discharge process, Sb_2_S_3_ undergoes lithiation via two different mechanisms, which are conversion reaction at ~ 1.4 V and alloying reaction at ~ 0.7 V. During the first charge process, the charge plateau occurs at ~ 1.1, 1.4, and 1.9 V, corresponding to the de-alloying process and the conversion reaction. During the second discharge process, the discharge plateau occurs at ~ 1.7 and 0.8 V. The charge/discharge voltage profile of the Sb_2_S_3_-150 electrode for the first discharge process is different from that for the second discharge process, indicating the presence of an activation process during the initial discharge process. Furthermore, the voltage plateaus of the charge/discharge profiles of the 50th cycle are perfectly retained. The Sb_2_S_3_-120 and Sb_2_S_3_-180 electrodes reveal similar charge/discharge voltage plateaus (Fig. S4), which are in agreement with the CV curves (Fig. S2).

The cycling performance of Sb_2_S_3_-120, Sb_2_S_3_-150, and Sb_2_S_3_-180 electrodes at a high current density of 1000 mA g^−1^ in the voltage range of 0.01–2.0 V (vs. Li/Li^+^) is verified in Fig. 4f, showing initial coulombic efficiencies of 68%, 69%, and 72%, respectively. It should be noted that the Sb_2_S_3_-150 electrode maintains a discharge capacity of 469 mAh g^−1^ with a coulombic efficiency of 98% after 100 cycles (90 mAh g^−1^ for Sb_2_S_3_-120 and 179 mAh g^−1^ for Sb_2_S_3_-180), manifesting superior cyclability. This is because the interior hollow structure can provide sufficient space to mitigate the volume change and pulverization, which is caused by the Li^+^ insertion/extraction [42–45]. For Sb_2_S_3_-120, the discharge capacity is higher than Sb_2_S_3_-150 and Sb_2_S_3_-180 in the first 30 cycles, but it decreases gradually and has a residual capacity of 90 mAh g^−1^ with a coulombic efficiency of 88% after 100 cycles. The higher discharge capacity of Sb_2_S_3_-120 is due to the nanowire size. During repeated insertion and extraction, the volume change and pulverization are the main causes attributed to the serious attenuation. For Sb_2_S_3_-180, the solid spherical structures affect the full contact between the electrolyte and active material, as well as the diffusion of Li^+^.

To further investigate the dynamics for lithium insertion and extraction of the Sb_2_S_3_-150 electrode, EIS measurements of Sb_2_S_3_-120, Sb_2_S_3_-150, and Sb_2_S_3_-180 electrodes were taken. The homologous 3D Nyquist plots are composed of an oblique line in the low-frequency region and a semicircle in the high-frequency region [46, 47]. The EIS data were analyzed by fitting with an equivalent electrical circuit model (Fig. 4i), and the fitted impedance data are listed in Table 1, where the oblique line is related to the Li^+^ diffusion within the active material, and the semicircle is applicable to the charge-transfer resistance (*R*_ct_) between the electrolyte and active material [48, 49]. It is clear that the fitting patterns are consistent with the experimental EIS data. Before cycling, the Sb_2_S_3_-120 sample shows a lower *R*_ct_ (Fig. 4g) than the Sb_2_S_3_-150 and Sb_2_S_3_-180 samples because the small size structure can provide additional reaction sites. After 50 cycles at a current density of 500 mA g^−1^ in the voltage range of 0.01–2.0 V (vs. Li/Li^+^), the *R*_ct_ of the Sb_2_S_3_-120 and Sb_2_S_3_-180 electrodes increases to 110 and 104 Ω, respectively (Fig. 4h). However, the *R*_ct_ of the Sb_2_S_3_-150 electrode only increases to 65 Ω, which is much less than that for the Sb_2_S_3_-120 and Sb_2_S_3_-180 electrodes, indicating that the 3D hollow microsphere structure of the Sb_2_S_3_-150 electrode can effectively enhance the diffusion ability of Li^+^ and facilitate charge transfer.

**Table 1:** The *R*_s_ and *R*_ct_ values of Sb_2_S_3_-120, Sb_2_S_3_-150, and Sb_2_S_3_-180 electrodes in LIBs

Sample	*R*_s_ (Ω)			*R*_ct_ (Ω)		
	Sb_2_S_3_-120	Sb_2_S_3_-150	Sb_2_S_3_-180	Sb_2_S_3_-120	Sb_2_S_3_-150	Sb_2_S_3_-180
Before cycle	0.001242	0.001629	0.002298	0.005954	0.007886	0.008348
After 50 cycles	16.83	3.135	3.997	110	65	104

Table 2 compares the synthesis method and electrochemical properties between hierarchical Sb_2_S_3_ hollow microspheres and other reported Sb_2_S_3_ in studies as anode materials for LIBs. As seen in the table, the hierarchical Sb_2_S_3_ hollow microspheres display superior electrochemical performance among unmodified Sb_2_S_3_ samples [16, 18, 50, 51]. Although the Sb_2_S_3_ obtained by the two-step oxidation–sulfuration method [18] achieved a higher discharge capacity of 548 mAh g^−1^ after 100 cycles, it should be noted that these data were acquired at a much lower current density of 100 mA g^−1^ and a wider voltage range (0.01–3.0 V). The Sb_2_S_3_-carbon composites, such as rGO-Sb_2_S_3_ [34] and Sb_2_S_3_/C [52], showed much better cycling stability than bare Sb_2_S_3_, which delivered the high specific capacity of 720 and 960 mAh g^−1^ after 50 and 30 cycles at a current density of 250 and 100 mA g^−1^, respectively. Nonetheless, the electrochemical properties at a high current density of 1000 mA g^−1^ were not mentioned. The Sb_2_S_3_/C prepared using the solid-state method [53] offered a high specific capacity of 600 mAh g^−1^ after 100 cycles, but this result was also obtained at a lower current density of 100 mA g^−1^. Sb_2_S_3_-graphite prepared by hydrothermal reaction and mechanical milling [54] exhibited an outstanding electrochemical performance of 656 mAh g^−1^ at a current density of 1000 mA g^−1^ after 100 cycles. Remarkably, the electrochemical performance of hierarchical Sb_2_S_3_ hollow microspheres in this work is comparable even with the previously reported Sb_2_S_3_-carbon composites. Meanwhile, it is widely accepted that composites with graphite or carbon can effectively improve the cyclability. In future work, the electrochemical properties of Sb_2_S_3_ hollow microspheres will be further improved by the addition of carbon-based materials.

**Table 2:** Comparison of the performance between hierarchical Sb_2_S_3_ hollow microspheres with other reported Sb_2_S_3_ in studies as anode materials for LIBs

Synthesis method	Samples	Voltage range (V)	Current density (mA g^−1^)	Capacity (mAh g^−1^) (cycle number)
Hydrothermal reaction [16]	Sb_2_S_3_	0.01–3.0	50	914(1)–175(10)
Two-step oxidation-sulfuration route [18]	Sb_2_S_3_	0.01–3.0	100	1187.2(1)–548(100)
Hydrothermal reaction [50]	Sb_2_S_3_	0–2.5	400	1049(1)–286(20)
Hydrothermal reaction [51]	Sb_2_S_3_	0.01–3.0	50	1070(1)–367(25)
Three-step wet-chemical synthesis method [34]	rGO-Sb_2_S_3_	0–2.5	250	880(1)–720(50)
Solvothermal treatment [52]	Sb_2_S_3_/C	0.01–2.5	100	1084(1)–960(30)
Hydrothermal reaction and mechanical milling [53]	Sb_2_S_3_-graphite	0.01–2.8	1000	720(1)–656(100)
Solid-state routes [54]	Sb_2_S_3_/C	0–2.5	100	757(1)–600(100)
Present work: hydrothermal reaction	Sb_2_S_3_	0.01–2.0	1000	1185(1)–469(100)

### Electrochemical Studies with Sodium-Ion Batteries

**Fig. 5:**
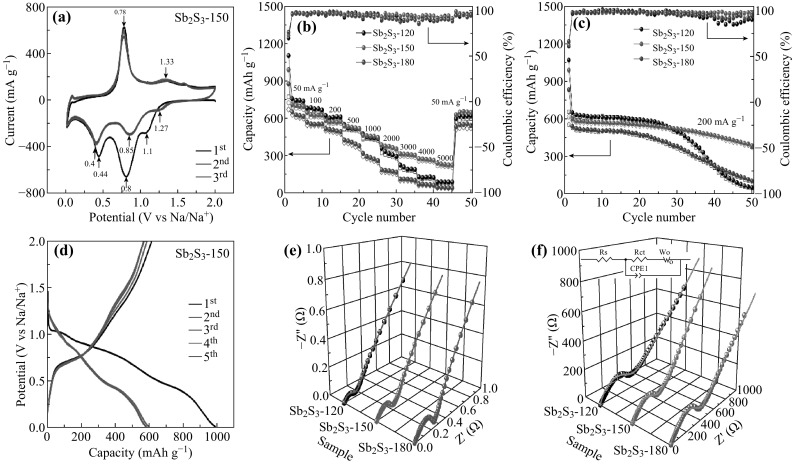
Excellent sodium-storage performance of as-prepared Sb_2_S_3_ electrodes. **a** CV curves of Sb_2_S_3_-150 in first three cycles with a scan rate of 0.1 mV s^−1^. **b** Rate capability of the Sb_2_S_3_-120, Sb_2_S_3_-150 and Sb_2_S_3_-180 at various current densities (increased from 50 to 5000 mA g^−1^). **c** Cycle performance of the Sb_2_S_3_-120, Sb_2_S_3_-150 and Sb_2_S_3_-180 electrodes at a current density of 200 mA g^−1^. **d** Charge/discharge voltage profiles of Sb_2_S_3_-150 electrode for the first five cycles at a current density of 200 mA g^−1^. **e** Three-dimensional Nyquist plots of Sb_2_S_3_-120, Sb_2_S_3_-150, and Sb_2_S_3_-180 electrodes at the open-circuit voltage. **f** Three-dimensional Nyquist plots of Sb_2_S_3_-120, Sb_2_S_3_-150, and Sb_2_S_3_-180 electrodes at a current density of 500 mA g^−1^ after 50 cycles (the inset is the equivalent circuit model)

Based on the excellent lithium-storage performance, it is important to attempt to extend the sodium-storage properties of the Sb_2_S_3_-120, Sb_2_S_3_-150, and Sb_2_S_3_-180 samples. The CV curves of hollow microsphere Sb_2_S_3_-150 electrodes for the initial three cycles at a scan rate of 0.1 mV s^−1^ in the voltage range of 0.01–2.0 V (vs. Na/Na^+^) are displayed in Fig. 5a. In the first cathodic scanning, the reduction peaks that are centered at 1.1, 0.8, and 0.44 V can be attributed to the conversion reaction between Sb_2_S_3_ and Na (Eq. 3), as well as the alloying reaction of Sb with Na (Eq. 4). The first anodic scanning gives two oxidation peaks that are stably centered at 0.78 and 1.33 V, resulting from the de-alloying reaction (reverse reaction of Eq. 4) and conversion reaction (reverse reaction of Eq. 3), respectively [15, 55]. During the subsequent scanning, the reduction peaks of the conversion reaction are located at 1.27 V, the alloying reaction are located at 0.4 and 0.85 V, and all of the peaks overlap very well, indicating that the hollow microsphere Sb_2_S_3_-150 sample has good stability.

3 $${\text{Conversion\,reaction:}} {\text{ Sb}}_{2} {\text{S}}_{3} + \, 6{\text{Na}}^{ + } + \, 6{\text{e}}^{ - } { \leftrightharpoons }3{\text{Na}}_{2} {\text{S }} + \, 2{\text{Sb}}$$

4 $${\text{Alloying\,reaction:}} \, 2{\text{Sb }} + \, 6{\text{Na}}^{ + } + \, 6{\text{e}}^{ - } { \leftrightharpoons }2{\text{Na}}_{3} {\text{Sb}}$$

The rate capabilities of Sb_2_S_3_-120, Sb_2_S_3_-150, and Sb_2_S_3_-180 electrodes were each assessed at current densities of 50, 100, 200, 500, 1000, 2000, 3000, 4000, and 5000 mA g^−1^ for five cycles in the voltage range of 0.01–2.0 V (vs. Na/Na^+^) (Fig. 5b). As with the lithium-storage performance, the Sb_2_S_3_-120 electrode also exhibits a higher discharge capacity at low current densities during the first few cycles. Sb_2_S_3_-180 shows the lowest specific capacity. The Sb_2_S_3_-150 electrode provides a discharge capacity of 990 mAh g^−1^ at a current density of 50 mA g^−1^. When the current density increases to 100, 200, 500, 1000, 2000, 3000, 4000, and 5000 mA g^−1^, the discharge capacity moderately decreases to 645, 582, 537, 463, 386, 314, 275, and 239 mAh g^−1^, respectively. When the current density decreases to 50 mA g^−1^, the reversible capacity can recover to 629 mAh g^−1^, indicating the distinguished rate capability of the Sb_2_S_3_-150 electrode, which may originate from the unique hierarchical hollow structure.

Figure 5c exhibits the cycling property of Sb_2_S_3_-120, Sb_2_S_3_-150, and Sb_2_S_3_-180 electrodes at a current density of 200 mA g^−1^, and the first discharge/charge capacities of 1069/656, 988/616, and 831/551 mAh g^−1^, with coulombic efficiencies of 61%, 62%, and 66%, respectively. The much lower initial specific capacity relative to that of LIB (1370/958, 1281/912, and 1058/767 mAh g^−1^) may be related to the slower ionic diffusion rate during the charging/discharging process in NIBs, which is due to the relatively large ionic radius of Na^+^ (102 pm) compared to that of Li^+^ (76 pm) [12, 56]. Interestingly, the Sb_2_S_3_-150 electrode exhibits good cycling stability and still provides a reversible capacity of 384 mAh g^−1^ with a coulombic efficiency of 98% after 50 cycles. For Sb_2_S_3_-120 and Sb_2_S_3_-180 electrodes, the capacities are stable for the initial 20 cycles, but decreases gradually, and has residual discharge capacities of 49 and 103 mAh g^−1^ (with coulombic efficiencies of 90% and 94%) after 50 cycles, respectively. These data verify that Sb_2_S_3_-150 with hollow microsphere structures can be a promising candidate anode material for NIBs.

The charge/discharge voltage profiles of the Sb_2_S_3_-150 electrode for the initial five cycles at a current density of 200 mA g^−1^ are shown in Fig. 5d. The first discharge voltage profile shows an obvious voltage platform located at ~1.1, 0.8, and 0.4 V, which is related to the conversion reaction and alloying reaction. The first charge–voltage profile exhibits a visible voltage platform located at ~0.8 and 1.3 V, corresponding to the de-alloying reaction and conversion reaction, respectively. In the subsequent cycling, the discharge voltage plateaus of the conversion reaction are located at ~1.2 and 0.8 V and the alloying reaction are located at 0.4 V, which is consistent with the CV results (Fig. 5a).

To further explain the prominent sodium-storage electrochemical behavior of Sb_2_S_3_-150 electrodes, EIS measurements of Sb_2_S_3_-120, Sb_2_S_3_-150, and Sb_2_S_3_-180 electrodes were taken. The inset of Fig. 5f shows the equivalent circuit model, which is in accordance with LIBs (Fig. 4i), and the fitted impedance data are recorded in Table 3. The *R*_ct_ value of the hollow microsphere Sb_2_S_3_-150 electrode is 0.21 Ω (0.11 Ω for Sb_2_S_3_-120, 0.245 Ω for Sb_2_S_3_-180) before cycling. Nevertheless, the *R*_ct_ value of the Sb_2_S_3_-150 electrode increases to 280 Ω at a current density of 500 mA g^−1^ after 50 cycles in the voltage range of 0.01–2.0 V (vs. Na/Na^+^). Meanwhile, the *R*_ct_ value of Sb_2_S_3_-120 and Sb_2_S_3_-180 electrodes increases to 360 and 350 Ω, which is much larger than the hollow microsphere-structured Sb_2_S_3_-150 electrode. This result infers that Sb_2_S_3_-150 compared to Sb_2_S_3_-120 and Sb_2_S_3_-180 electrodes has a relatively good electrochemical performance.

**Table 3:** The *R*_s_ and *R*_ct_ values of Sb_2_S_3_-120, Sb_2_S_3_-150, and Sb_2_S_3_-180 electrodes in NIBs

Sample	*R*_s_ (Ω)			*R*_ct_ (Ω)		
	Sb_2_S_3_-120	Sb_2_S_3_-150	Sb_2_S_3_-180	Sb_2_S_3_-120	Sb_2_S_3_-150	Sb_2_S_3_-180
Before cycle	0.04429	0.04819	0.05202	0.11	0.21	0.245
After 50 cycles	6.521	5.305	6.455	360	280	350

Table 4 compares the synthesis method and electrochemical properties between hierarchical Sb_2_S_3_ hollow microspheres and other reported Sb_2_S_3_ in studies as anode materials for NIBs. As can be seen, the hierarchical Sb_2_S_3_ hollow microspheres in this work present excellent electrochemical performance compared to the reported Sb_2_S_3_ microtubes [20]. In addition, it is even comparable with the reported Sb_2_S_3_ synthesized reflux process [14] and a-Sb_2_S_3_ obtained using the polyol-mediated process [57], which was obtained at a lower current density of 50 mA g^−1^. Nevertheless, compared to that of the previously reported Sb_2_S_3_-carbon composites, such as Sb_2_S_3_/C [58], rGO/Sb_2_S_3_ prepared using the solution-based synthesis technique [19], Sb_2_S_3_/RGO [47], Sb_2_S_3_/SGS synthesized using a modified Hummers’ method [55], Sb_2_S_3_ in a P/C composite obtained by a mechanochemical process with heat treatment [59], Sb_2_S_3_@C [60], MWNTs@Sb_2_S_3_@PPy prepared using the two-step wet-chemical synthesis method [61], and ZnS-Sb_2_S_3_@C synthesized by the microthermal solvothermal sulfidation process [62], the electrochemical performance of hierarchical Sb_2_S_3_ hollow microspheres in this work is lower. It can be deduced that Sb_2_S_3_ as an anode material for NIBs possesses more serious capacity fading, and the adding carbon-based materials can enhance the cycling stability of Sb_2_S_3_ obviously. The capacity fading can be attributed to the relatively large ionic radius of Na^+^ compared to that of Li^+^, which gives rise to a series of problems, such as large variations in volume, severe pulverization, and slow ionic diffusion rate during the repeated charging/discharging process in NIBs [6, 10, 63]. These issues cause poor reversibility and low rate capacity. So, it is important to improve the electrochemical properties of Sb_2_S_3_ hollow microspheres in this work by coating or developing composites using carbon-based materials, making the development of high-performance Sb_2_S_3_ anode materials for NIBs challenging.

**Table 4:** Comparison of the performance between hierarchical Sb_2_S_3_ hollow microspheres with other reported Sb_2_S_3_ in studies as anode materials for LIBs

Synthesis method	Samples	Voltage range (V)	Current density (mA g^−1^)	Capacity (mAh g^−1^) (cycle number)
One-step hydrothermal method [20]	Sb_2_S_3_	0–1.5	100	910(1)–201(20)
Reflux process [14]	Sb_2_S_3_	0.01–2.0	50	970(1)–835.3(50)
Polyol-mediated process [57]	a-Sb_2_S_3_	0.01–2.5	50	650(1)–512(100)
Hydrothermal reaction [58]	Sb_2_S_3_/C	0–2.0	100	1200(1)–570(100)
Solution-based synthesis technique [19]	rGO/Sb_2_S_3_	0–2.0	50	660(1)–670(50)
Hydrothermal reaction [47]	Sb_2_S_3_/RGO	0.005–3.0	50	1170(1)–581.2(50)
A modified Hummers’ method [55]	Sb_2_S_3_/SGS	0.01–2.5	2000	720(1)–524.4(900)
Mechanochemical process with heat treatment [59]	Sb_2_S_3_ in P/C	0.005–2.0	50	818(1)–611(100)
Hydrothermal reaction [60]	Sb_2_S_3_@C	0.01–2.5	100	1066(1)–730(100)
Two-step wet-chemical synthesis method [61]	MWNTs@Sb_2_S_3_@PPy	0–2	100	870 (1)–500(85)
Microthermal solvothermal sulfidation process [62]	ZnS-Sb_2_S_3_@C	0.01–1.8	100	1660(1)–630(120)
Present work: hydrothermal reaction	Sb_2_S_3_	0.01–2.0	200	988(1)–384(50)

## Conclusion

In summary, a straightforward and inexpensive hydrothermal method was employed to prepare Sb_2_S_3_ with a hollow microsphere architecture. Such a unique structure combines the merit of the nanometer size and self-assembly capabilities, achieving a large contact surface area between the electrolyte and active material, short diffusion path for the Li^+^/Na^+^ and electron, and additional space to accommodate volume expansion caused by Li^+^/Na^+^ insertion/extraction during the repeated charging/discharging process. The resultant Sb_2_S_3_ hollow microspheres exhibit desirable electrochemical properties, such as high capacity and remarkable reversibility, when used as an anode material in LIBs. A large discharge capacity of 458 mAh g^−1^ at a current density of 1000 mA g^−1^ after 100 cycles is achieved. The Sb_2_S_3_ hollow microspheres also reveal remarkable rate capability and cycling performance as anode material for NIBs, keeping a reversible discharge capacity of 384 mAh g^−1^ at a current density of 200 mA g^−1^ after 50 cycles. These prominent lithium/sodium-storage performance enable the hollow microsphere Sb_2_S_3_ to become competitive among many anode materials reported for LIBs/NIBs. In the future, the cycle performance can be further improved by using graphene compounds (or other conducting polymers).

## Electronic supplementary material

Below is the link to the electronic supplementary material.

Supplementary material 1 (PDF 658 kb)
