# Supplementary material for: Template-Free Synthesis of Sb2S3 Hollow Microspheres as Anode Materials for Lithium-Ion and Sodium-Ion Batteries
Source: Nanomicro Lett. 2017 Oct 31;10(1):12. doi: 10.1007/s40820-017-0165-1 (PMC6199062; doi:10.1007/s40820-017-0165-1)
Supplement: Supplementary file 1 — Supplementary material 1 (PDF 658 kb) [file 40820_2017_165_MOESM1_ESM.pdf]

Supporting Information for

Template-Free Synthesis of Sb_2S_3 Hollow Microspheres as Anode Materials for Lithium-Ion and Sodium-Ion Batteries

Jianjun Xie[1], Li Liu[1, 2, *], Jing Xia[1], Yue Zhang[1], Min Li[1], Yan Ouyang[1], Su Nie[1], Xianyou Wang[1]

[1]National Base for International Science & Technology Cooperation, National Local Joint Engineering Laboratory for Key materials of New Energy Storage Battery, Hunan Province Key Laboratory of Electrochemical Energy Storage and Conversion, School of Chemistry, Xiangtan University, Xiangtan 411105, People's Republic of China

[2]Key Laboratory of Advanced Energy Materials Chemistry (Ministry of Education), Nankai University, Tianjin 300071, People's Republic of China

*****Corresponding author. E-mail: liulili1203@126.com

Tel.: +86-731-58292206; Fax: +86-731-58292477

Figures

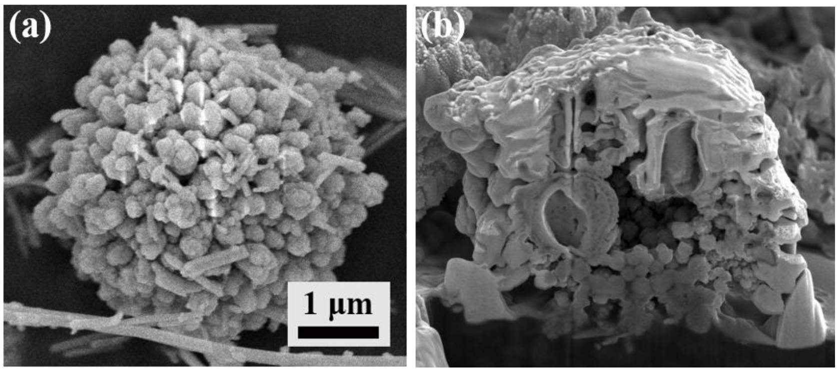

Fig. S1 a Random selected a particle of Sb_2S_3-150 sample. **b** After dissected Sb_2S_3-150 particle

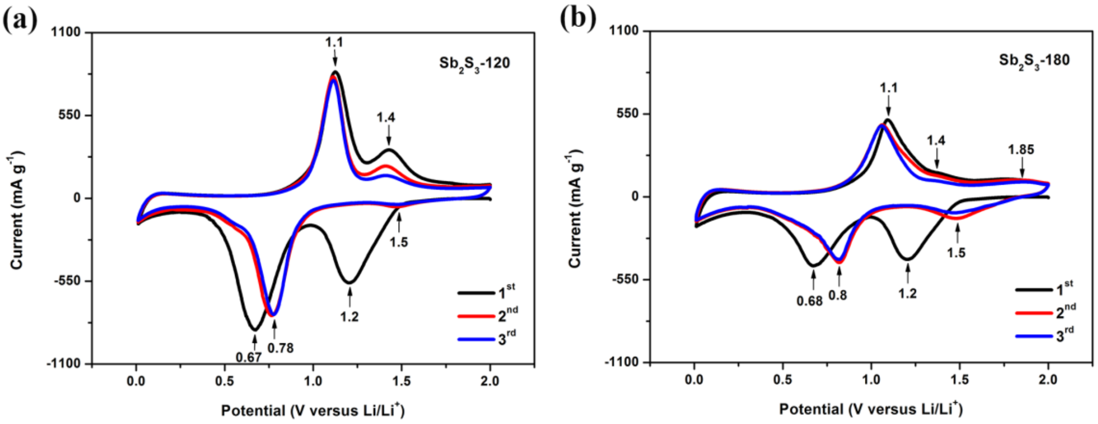

Fig. S2 CV curves of **a** Sb_2S_3-120 and **b** Sb_2S_3-180 electrodes for the first three cycles at a scan rate of 0.1 mV s^{-1} in LIBs

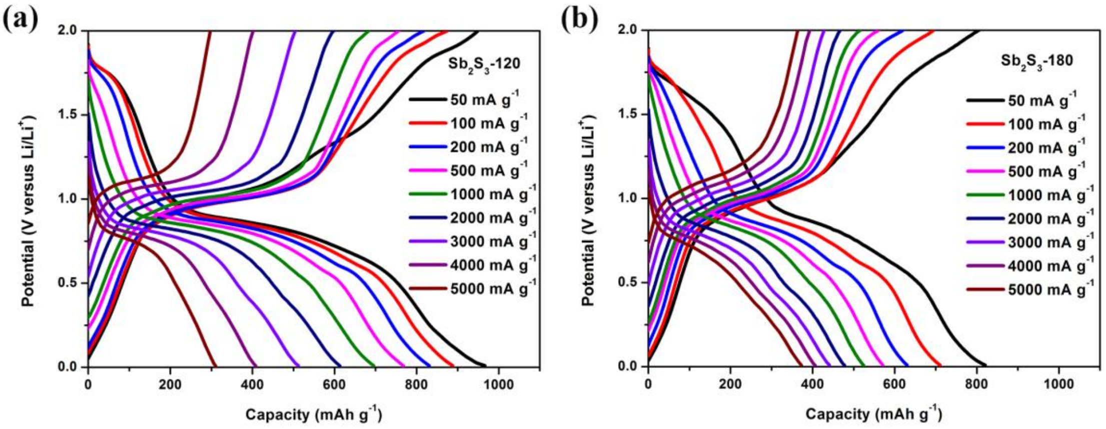

Fig. S3 Selected charge/discharge voltage profiles of **a** Sb_2S_3-120 and **b** Sb_2S_3-180 electrodes at different current densities in LIBs

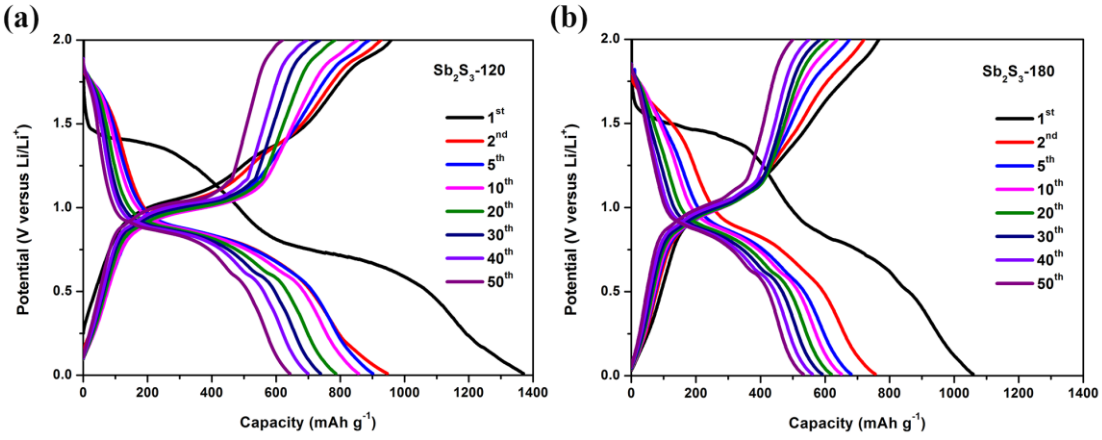

Fig. S4 Selected charge/discharge voltage profiles of **a** Sb_2S_3-120 and **b** Sb_2S_3-180 electrodes at a current density of 200 mA g^{-1} in LIBs
